# The Ebola Virus VP30-NP Interaction Is a Regulator of Viral RNA Synthesis

**DOI:** 10.1371/journal.ppat.1005937

**Published:** 2016-10-18

**Authors:** Robert N. Kirchdoerfer, Crystal L. Moyer, Dafna M. Abelson, Erica Ollmann Saphire

**Affiliations:** 1 Department of Immunology and Microbial Sciences, The Scripps Research Institute, La Jolla, California, United States of America; 2 Skaggs Institute for Chemical Biology, The Scripps Research Institute, La Jolla, California, United States of America; Thomas Jefferson University, UNITED STATES

## Abstract

Filoviruses are capable of causing deadly hemorrhagic fevers. All nonsegmented negative-sense RNA-virus nucleocapsids are composed of a nucleoprotein (NP), a phosphoprotein (VP35) and a polymerase (L). However, the VP30 RNA-synthesis co-factor is unique to the filoviruses. The assembly, structure, and function of the filovirus RNA replication complex remain unclear. Here, we have characterized the interactions of Ebola, Sudan and Marburg virus VP30 with NP using *in vitro* biochemistry, structural biology and cell-based mini-replicon assays. We have found that the VP30 C-terminal domain interacts with a short peptide in the C-terminal region of NP. Further, we have solved crystal structures of the VP30-NP complex for both Ebola and Marburg viruses. These structures reveal that a conserved, proline-rich NP peptide binds a shallow hydrophobic cleft on the VP30 C-terminal domain. Structure-guided Ebola virus VP30 mutants have altered affinities for the NP peptide. Correlation of these VP30-NP affinities with the activity for each of these mutants in a cell-based mini-replicon assay suggests that the VP30-NP interaction plays both essential and inhibitory roles in Ebola virus RNA synthesis.

## Author Summary

Filoviruses use a system of proteins and RNA to regulate viral RNA genome transcription and replication. Here, we have determined crystal structures and the biological functions of the protein complex formed by the filovirus transcriptional activator, VP30, and the core component of the nucleocapsid machinery, NP. The complex of these two essential players represses Ebola virus RNA synthesis and may have played a role in the evolution of filoviruses to tune viral RNA synthesis activity to a level ideal for infection. This interaction is conserved across the filoviruses and may provide an opportunity for therapeutic development.

## Introduction

Filoviruses such as Ebola (EBOV) and Marburg viruses (MARV) are nonsegmented negative-sense RNA viruses that can cause deadly hemorrhagic fevers with up to 90% fatality [1]. The impact of EBOV is highlighted by the recent outbreak in West Africa involving over 28,000 cases and claiming more than 11,000 lives [2]. Key to the viral life cycle are the components of the viral nucleocapsid. The nucleocapsids of all nonsegmented negative-sense RNA viruses carry a viral RNA-dependent, RNA polymerase (L), a phosphoprotein polymerase co-factor (P or VP35) and a nucleoprotein (N or NP), which encapsidates the viral genome. In the *Mononegavirales* order of viruses, L and NP interact through the phosphoprotein to carry out viral RNA synthesis. Filoviruses are unusual among mononegaviruses in that they encode an additional nucleocapsid component, VP30.

VP30 is a multifunctional protein and acts as a transcriptional activator [3]. EBOV VP30 promotes read-through of an RNA hairpin in the NP open reading frame to enhance viral transcription [4]. EBOV VP30 also assists stop-start transcription at gene junctions to promote transcription of downstream genes [5]. The N-terminal portion of VP30 contains phosphorylation sites, a zinc-binding site, and a RNA-binding site. Phosphorylation in the N-terminal region regulates association of EBOV VP30 with the nucleocapsid and alters the balance of viral transcription and RNA replication [5–8]. Binding of zinc is important for its transcriptional enhancement activity, and capacity to bind RNA may facilitate the interaction of VP30 with the viral genome [9, 10]. The C-terminal domain of VP30 (CTD, amino acids 139–288) forms a conserved dimer of two globular, α-helical domains assembled by the extension of one α-helix from each protomer across the dimer interface to contact the adjacent protomer [11, 12]. EBOV VP30 binds directly to nucleocapsid components L and NP [13–15]. Hartlieb, *et al*. determined that it is the VP30 CTD that contains the binding site for the viral NP [11].

The uniqueness of filovirus VP30 among nonsegmented negative-sense RNA viruses has left many unanswered questions concerning its role in viral RNA transcription and replication. Despite available functional data, very little is known about the mechanisms by which VP30 carries out these functions or interacts with other viral components. Here, we have mapped the filovirus VP30-binding site on NP and determined crystal structures of the complexes for EBOV and MARV. We have used these structures to explore the function of the VP30-NP interaction using rational mutagenesis and mini-replicon assays. This work shows the importance of the EBOV VP30-NP interaction for viral RNA synthesis and provides clues to understand how this interaction contributes to the regulation of the filovirus nucleocapsid complex.

## Results

### The VP30 C-Terminal Domain Binds a Peptide in the NP C-Terminal Region

We first demonstrated that the EBOV VP30 CTD binds to the NP C-terminal portion (360–739). The N-terminal half of NP (a.a. 1–390) forms an ordered structure and plays important roles in NP-NP oligomerization and RNA binding [16, 17]. In contrast, the C-terminal half of NP (residues 391–739) is mostly disordered, although a short folded domain exists at its C terminus (residues 645–739) [18]. Hypothesizing that VP30 may recognize a portion of NP within the C-terminal half of NP, we carried out isothermal titration calorimetry (ITC) using the VP30 CTD, which Hartlieb et al. has previously shown interacts with NP [11] and the C-terminal portion of NP. This experiment demonstrated that the EBOV VP30 CTD binds to the NP C-terminal portion with a K_D_ of 21.3 ± 4.5 μM.

**Fig 1:**
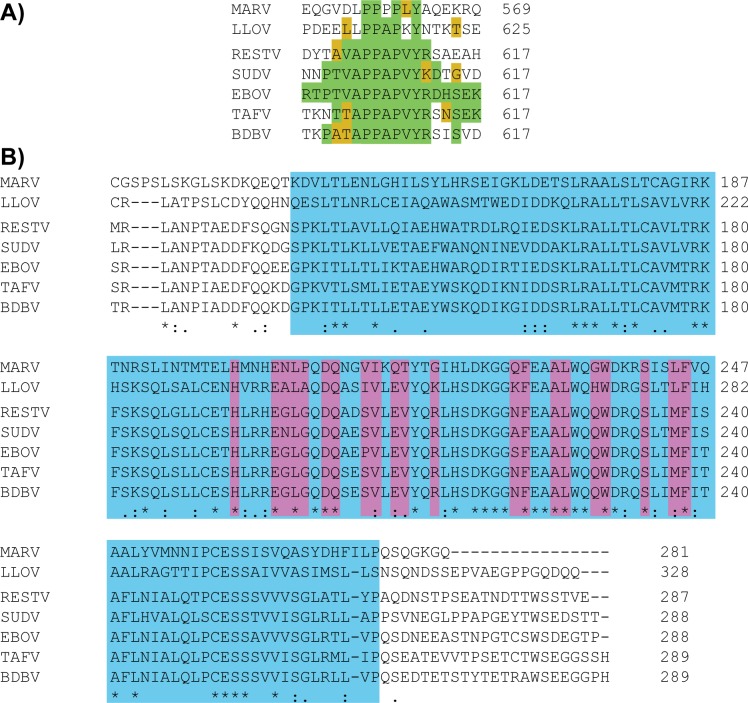
Conservation of NP and VP30 interactions. A) Sequences of filovirus NP 600–617 (EBOV numbering) were aligned with Clustal Omega [19]. Residues identical to EBOV are colored green and similar residues are in yellow. B) Sequences of filovirus VP30 C-terminal region were aligned with Clustal Omega [19]. Those amino acids of the VP30 CTD that are visible in the EBOV VP30-NP complex crystal structure are colored blue. VP30 amino acids within 5 Å of EBOV NP 602–614 are indicated in purple.

We next used biolayer interferometry (BLI) to fine map the VP30-binding site on Ebola virus NP. We generated several biotinylated EBOV NP peptides: 360–420, 419–496, 560–617 and 644–739 based on sequence conservation among the five ebolavirus species. We immobilized the EBOV NP peptides on streptavidin-coated biosensors and bound them to EBOV VP30 CTD. Due to difficulties in curve fitting and the dimeric VP30 CTD likely acting as a multivalent binder, these BLI experiments were performed qualitatively. VP30 CTD bound to NP 560–617, but not to any of the other NP peptides. We further used a series of shorter overlapping NP peptides: 560–580, 580–600, 600–617, 560–600 and 580–617, to define the VP30-binding site more narrowly. Only EBOV NP 580–617 and 600–617 bound to VP30 CTD (S1 Fig). This result strongly suggested that the primary binding site for EBOV VP30 CTD lies within NP 600–617. This region of NP contains a stretch of amino acids that is conserved across the ebolaviruses and shares homology with cuevaviruses and marburgviruses (Fig 1A).

To quantitatively assess the molecular interaction of EBOV NP 600–617 with VP30 CTD, we performed ITC using NP 600–617 attached to T4 lysozyme as a carrier protein. The fusion of NP to lysozyme permits the preparation of this protein recombinantly, the accurate measurements of protein concentration using UV absorbance and the efficient dialysis prior to ITC, which would have otherwise been complicated for a short peptide. We observed no binding of VP30 CTD to T4 lysozyme alone (S2 Fig). EBOV VP30 CTD binds to the NP 600–617 peptide with a K_D_ of 5.69 ± 0.04 μM, a modestly higher affinity than for NP 360–617, confirming the findings from the BLI experimentation that this NP peptide is sufficient for VP30 binding.

**Fig 2:**
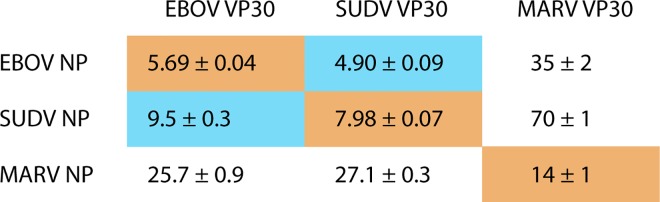
Cross-species binding of filovirus VP30 and NP. ITC experiments were carried out using EBOV, SUDV or MARV VP30 CTD (ebolavirus 139–288 or Marburgvirus 146–281) and EBOV, SUDV or MARV NP peptide (ebolavirus 600–617 or Marburg virus 552–569) fused to T4 lysozyme. Equilibrium dissociation constants (K_D_s) are presented in micromolar and are the average of three replicates. Species-matched pairs are indicated in orange and intra-genus pairs in blue.

Because the EBOV NP 600–617 peptide is highly conserved across ebolaviruses and has homology across filoviruses, we performed cross-species binding experiments using ebolavirus NPs 600–617 and MARV NP 552–569 with VP30 CTDs (MARV VP30 146–281) using ITC (Fig 2). Significant cross-reactivity is noted, especially among the ebolaviruses. Among the ebolavirus NPs, that of Ebola virus (EBOV) appears to be a stronger binder of the ebolavirus VP30s. Among the VP30s, that of Sudan ebolavirus (SUDV) is a stronger binder of the ebolavirus NPs.

MARV VP30 recognizes its own NP peptide with reasonable affinity, 14 ± 1 μM. Cross-reactivity across genera also occurs. However, this binding is weaker than the binding observed within members of the ebolavirus genus, as expected from the weaker conservation of the MARV NP peptide and VP30 CTD to those of the ebolaviruses. These results are supported by previous experiments demonstrating that MARV VP30 was able to support transcription, albeit with much lower efficiency, when exchanged for EBOV VP30 in an EBOV mini-replicon reporter assay [3]. These data show that the interaction of each VP30 CTD with its NP 600–617 is conserved across the filoviruses.

**Fig 3:**
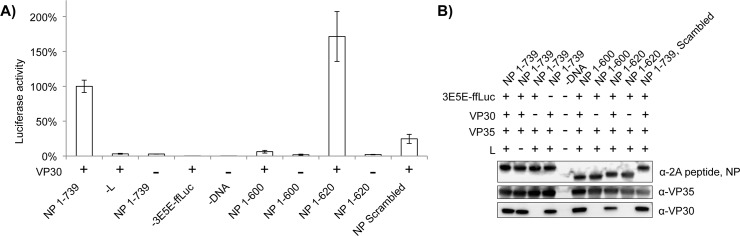
The NP 600–620 peptide is essential for mini-replicon activity. Mammalian cells were transfected with plasmids encoding the Ebola virus RNA synthesis components NP, VP35, VP30 and L, along with a model viral mini-replicon encoding firefly luciferase. Full-length NP was exchanged with either NP 1–600 or 1–620 in the presence or absence of VP30. A full-length NP mutant, in which the conserved residues from 605–612 had been scrambled to 605-PVARPYAP-612, was also included. Negative controls for the assay included transfections lacking the viral polymerase (-L) or the mini-replicon RNA (-3E5E-ffLuc) and a no-DNA mock transfection (-DNA). A) Data is shown as the percent luciferase activity compared to the wild-type positive control (left-most sample) and is the average of three independent experiments. B) Western blot for NP, VP35 and VP30 of cleared cellular lysates transfected with the mini-replicon components. All truncated and mutant NPs expressed at levels similar to wild-type NP.

To further confirm our mapping of this interaction on the viral NP and show its importance in RNA synthesis, we performed viral mini-replicon assays. In this assay, cells are transfected with plasmids expressing the minimal Ebola virus protein components for RNA synthesis: NP, VP35, VP30 and L, along with a plasmid expressing a negative-sense RNA encoding firefly luciferase flanked by Ebola virus leader and trailer sequences [20]. In this system, proper assembly and function of the RNA synthesis machinery leads to expression of the reporter. Leung, et al. has previously shown that when a truncated NP 1–600 is used in place of the full-length NP (1–739), that no mini-genome activity is observed in this assay [21]. Conversely, Watanabe, *et al*. has demonstrated that NP 1–600 is sufficient for reporter production in similar assays [17]. Our own results with NP 1–600 indicate a complete loss of reporter activity consistent with the results of Leung, *et al*. [21] (Fig 3). The NP 1–600 truncation has been shown to form the helical oligomers typical of non-segmented negative-sense RNA viruses, to form nucleocapsid-like structures in the presence of VP35 and VP24 [17], and to contain the NP RNA-binding site [16, 22]. The NP 1–600 truncation, however, excludes our newly identified VP30-binding site. Increasing the length of the NP protein construct to residues 1–620 restores luciferase activity validating our VP30-binding site on NP (residues 600–617). As a further check on the importance of this site within NP, we exchanged the full-length, wild-type NP for an NP in which the conserved amino acids within 600–617 were scrambled (605-PVARPYAP-612). The scrambled mutant NP showed large reductions in binding to the VP30 CTD *in vitro* by ITC (S2 Fig) and in luciferase activity in the mini-replicon assay (Fig 3). The *in vitro* binding data show that the NP 600–617 is necessary for VP30 binding and results from the coupled transcription/replication mini-replicon assays show that reporter activity is dependent on the NP-VP30 interaction.

### Crystal Structure of VP30 CTD Bound to NP 600–617

To understand the molecular basis for the VP30-NP interaction, we sought to obtain high resolution X-ray crystallographic data for the complex. The rather low affinity between VP30 CTD and NP 600–617 precludes the co-purification of the complex. Presumably, when both VP30 and NP are full-length and oligomeric, multivalent binding greatly increases the avidity of this interaction. However, for crystallographic studies we used truncated viral proteins to reduce conformational heterogeneity that may impede crystal formation. To overcome the low-affinity of the interaction, we expressed the EBOV NP peptide (600–627) as a fusion to the N terminus of EBOV VP30 CTD, and the MARV NP peptide (552–579) as a fusion to the N terminus of MARV VP30 CTD (146–281). These portions of NP include the identified VP30-interaction sites, as well as an additional ten amino acids that act as linkers to the VP30 CTDs. Both protein fusions crystallized and X-ray diffraction data were collected. The EBOV NP-VP30 fusion protein diffracted to 2.20 Å with ten protomers in the asymmetric unit, while the MARV NP-VP30 construct diffracted to 3.25 Å with eight protomers in the asymmetric unit (Table 1). Each of the VP30 CTD protomers in both complex structures is bound to a NP peptide.

**Table 1:** X-ray diffraction data collection and processing statistics.

	EBOV NP-VP30	MARV NP-VP30
PDB ID	5T3T	5T3W
Data Collection		
Space Group	P 1 2_1_ 1	P 3_1_ 2 1
a b c (Å)	85.9, 116.8, 95.0	106.1, 106.1, 375.6
α β γ (°)	90.0, 108.5, 90.0	90.0, 90.0, 120.0
Wavelength (Å)	1.03320	1.75020
Resolution (Å)	50–2.20 (2.24–2.20)	50–3.25 (3.38–3.25)
Total reflections	266,450 (12,605)	761,914 (85,995)
Unique-reflections	89,012 (4,245)	39,861 (4,402)
Redundancy	3.0 (3.0)	19.1 (19.5)
Completeness (%)	98.6 (93.0)	100.0 (100.0)
<I/σ(I)>	7.3 (1.4)	15.9 (1.1)
R_merge_	0.12 (0.95)	0.11 (2.96)
R_pim_	0.09 (0.66)	0.03 (0.69)
CC_1/2_	0.99 (0.57)	1.00 (0.61)
Refinement		
Resolution	50–2.20 (2.26–2.20)	50–3.25 (3.33–3.25)
R_work_	0.22 (0.33)	0.21 (0.41)
R_free_	0.25 (0.38)	0.24 (0.42)
No. of atoms	11,795	8,624
Protein	10,805	8,624
Solvent and ions	990	0
R.m.s. deviations from ideal geometry		
Bond lengths (Å)	0.013	0.007
Bond angles (°)	1.58	1.142
Average temperature factors (Å^2^)		
Wilson plot	28.2	126.6
VP30	43.0	145.1
NP peptide	49.0	158.5
Solvent and ions	49.5	-
Ramachandran angles (%)		
Favored	98.3	97.6
Allowed	1.7	2.4
Outliers	0.0	0.0

Amongst the copies of EBOV VP30 in the asymmetric unit, the N-terminal-most amino acid visible is 140 or 141 while the visible C-terminus is amino acid 265 or 266. Amongst copies of NP, visible residues range from 602 or 603 to 612 or 614. All of the EBOV NP peptides bind to VP30 in an identical fashion. The high resolution of the data allows visualization of many solvent molecules, some near the interfaces of NP and VP30 as well as between VP30 protomers. There also appears to be a clustering of sulfate ions from the crystallization condition near the VP30 dimer interfaces. This clustering reflects the presence of several basic residues near the VP30 dimer interfaces.

**Fig 4:**
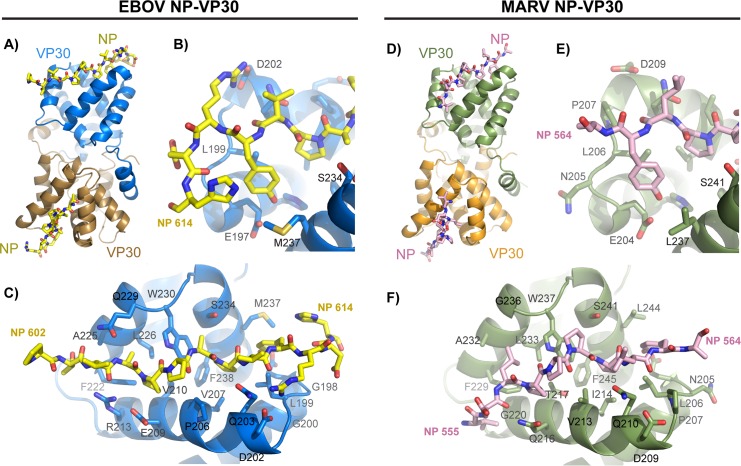
Filovirus NP binds to VP30 in a shallow hydrophobic cleft. A) In the crystal structure, EBOV VP30 CTD forms a dimer of globular domains (cartoon). NP 602–614 binds in a shallow cleft on the VP30 globular domain distal to the dimeric interface (sticks). B) and C) The NP-binding site on VP30 is mostly hydrophobic. D) MARV VP30 CTD also forms a dimer of globular domains and binds MARV NP peptide in the same location as EBOV. E) and F) The MARV NP binding site is also hydrophobic and contains many similar interactions with EBOV. The MARV NP peptide turns N-terminal of P558 to present a different conformation.

Amino acids from the EBOV NP peptide contacting VP30 are primarily those that are highly conserved across ebolaviruses. NP contacts VP30 on the EBOV VP30 globular C-terminal domain, distal to the VP30 dimer interface (Fig 4A). The NP-VP30 interface is primarily hydrophobic. Very few NP side chains make hydrogen bonds to VP30. However, NP R612 participates in a salt bridge to VP30 D202 and NP Y611 forms a hydrogen bond to VP30 E197 (Fig 4B and 4C). The NP peptide makes several hydrogen bonds using its main-chain atoms to the VP30 side chains of Q203, R213, Q229 and W230.

Analysis of the EBOV VP30-NP complex structure with a sequence alignment of ebolavirus VP30s (Fig 1B) shows that of the 21 VP30 amino acids within 5 Å of the NP peptide, 18 are conserved across ebolaviruses. Of the three that show some polymorphism, proline at VP30 position 206 is conserved among variants of EBOV, but is a serine in other species of the ebolavirus genus (SUDV, Reston, Bundibugyo and Taï Forest ebolaviruses). Glycine at EBOV VP30 position 198 is an asparagine in SUDV, and serine at position 221 is instead an alanine or asparagine in other ebolaviruses. Polymorphisms at positions 198 or 221 are not predicted to have a strong impact on NP-binding, as these residues are peripheral to the binding site. Of the NP residues, the eight contained within 605-APPAPVYR-612 are strictly conserved across all ebolaviruses with the exception of R612, which is a lysine in SUDV (Fig 1A). Amino acids in ebolavirus NPs flanking this sequence are poorly conserved, but appear not to make intimate contacts.

In the MARV VP30-NP complex structure, NP amino acids 555 to 564 and VP30 147 to 273 are visible in all of the protomers. The overall structure of the MARV VP30 CTD is remarkably similar to that of EBOV VP30 CTD despite sharing only 35% sequence identity across the CTD regions visualized in the crystal structures (Fig 4D). The conformation of the bound MARV NP peptide is also remarkably similar to that of EBOV especially across the NP proline-rich region (Fig 4E and 4F). The conserved binding mode of these NP peptides is supported by the ITC binding data above where cross-genus binding is possible, albeit with lower affinity than the species- and genus-matched interaction pairs. However, the conformation of the NP peptide is more divergent between EBOV and MARV N-terminal to the proline-rich region. In this region of the NP-VP30 complex, there is evidence for divergence and the evolution of compensatory mutations. In EBOV NP, A605 packs against the EBOV VP30 207–229 α-helix, but in MARV NP, the presence of amino acid L557 at the equivalent position turns the NP backbone towards the C-terminal half of the MARV VP30 208–223 α-helix. The altered NP backbone conformation gives rise to compensatory mutations including E209Q and R213G (EBOV to MARV, EBOV numbering). The E209Q allows the MARV NP main chain to hydrogen bond at this position and avoid an electrostatic clash with the NP main-chain carbonyls. The R213G change allows the MARV NP peptide to avoid a clash with this side chain. In addition, other amino acid differences make the MARV and EBOV proteins less apt cross-genus binding partners. These MARV amino acid differences include the lack of a basic residue in NP at 564 (EBOV NP R612) to form a salt bridge with VP30 D209 (EBOV VP30 D202), as well as VP30 G236 (EBOV VP30 Q229), which can no longer make hydrogen-bonding contacts to the NP backbone. The lack of these interactions in MARV NP and VP30 may result in the modestly lower affinity observed in ITC experiments and make MARV NP and VP30 poor binding partners for their ebolavirus protein counterparts.

**Fig 5:**
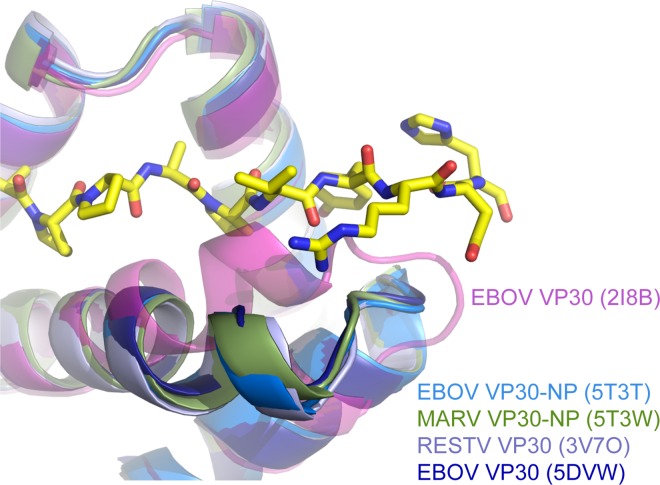
A previously determined EBOV VP30 CTD structure clashes with the NP peptide. Previously determined VP30 CTD structures as well as the VP30 CTD-NP peptide structures described here were structurally aligned using PyMol [27]. The EBOV NP peptide (602–614) bound to VP30 CTD is in yellow and shown as sticks. NP peptide binding is incompatible with the conformation of the VP30 CTD in 2I8B.pdb [11].

Comparison of the EBOV and MARV VP30-NP complex structures presented here with the first structure of the EBOV VP30 C-terminal domain (2I8B.pdb [11]) shows a small conformational difference in the VP30 α-helix composed of residues 201–216, where the 2I8B.pdb conformation of this α-helix would clash with the NP peptide in our complex structures (Fig 5). To explore a potential conformational change experimentally, we used ITC to measure the heat capacity change upon binding of EBOV VP30 to NP (ΔC_p_) and the extrapolated temperature at which the entropy of the binding event is zero (T_S_). We used these two values to calculate the buried non-polar surface area upon binding and the number of amino acids rigidified upon binding [23, 24] (S3 Fig). The buried non-polar surface area calculated from the ITC experiments is 520 Å^2^, which is similar to the 620 Å^2^ of total buried surface area observed in the EBOV NP-VP30 crystal structure. The calculated number of amino acids rigidified upon binding is approximately ten amino acids, similar to the 10–13 amino acids of NP observed in the complex structure. Because this region of NP is predicted to be disordered [25], the observed rigidification can likely be attributed to the NP peptide. The buried non-polar surface area and amino acid rigidification data suggest that the binding site on VP30 is pre-formed. To reconcile this finding with the differences observed between 2I8B.pdb and the structures presented here, we further compared our structures to the Reston ebolavirus (RESTV) VP30 CTD (3V7O.pdb [12]) and a more recently determined structure of the EBOV VP30 CTD (5DVW.pdb [26]). In both of these un-complexed structures, the VP30 α-helices composed of residues 201–216 align closely to the EBOV and MARV VP30-NP complexes, supporting our finding of a preformed NP-binding site on VP30. A closer examination of 2I8B.pdb reveals that the modest difference in conformation is likely induced by a neighboring protomer in the crystal lattice.

### Modulation of the EBOV VP30-NP Interaction

With crystal structures of the VP30-NP complexes in hand, we designed targeted EBOV VP30 mutations to alter the affinity of NP for VP30. To strongly impact the NP-VP30 interaction, we mutated our structure-selected amino acid positions to arginine, with the exception of a partially buried tryptophan residue, which we conservatively mutated to phenylalanine (W230F). We expressed the mutant VP30 CTD proteins recombinantly and tested their ability to interact with the NP peptide using ITC. Despite the high concentrations of protein used in these assays, no binding was observed for VP30 E197R, W230F, Q203R or S234R, indicating that the equilibrium dissociation constants for these mutant dimeric VP30 CTDs with monomeric NP peptide is likely greater than 200 μM. The negative impact of these VP30 CTD mutants on NP peptide binding validates the NP-VP30 interaction sites observed in the crystal structures. Two of our EBOV VP30 CTD mutants showed a reduced, yet measurable, affinity for NP peptide: D202R and Q229R (Table 2). Finally, a single mutant, P206R, showed an increased affinity for the NP peptide. P206 lies in EBOV VP30 α-helix 201–216. Despite being conserved in Ebola viruses, the presence of a proline residue in the middle of a helix suggests that this proline may destabilize the helical conformation of this region leading to a non-ideal NP peptide-binding site. This notion is further supported by the presence of a serine at position 206 in other ebolaviruses and a valine in MARV. Indeed, SUDV VP30 recognizes EBOV NP with higher affinity than does EBOV VP30, despite being otherwise highly conserved across the NP-binding site (Figs 1B and 2).

**Table 2:** EBOV VP30 mutations to the NP-binding site alter the affinity of the interaction.

EBOV VP30	K_D_ (μM)
Wild-type	5.69 ± 0.04
E197R	>200
D202R	27.2 ± 0.8
Q203R	>200
P206R	1.92 ± 0.07
Q229R	48.4 ± 1.6
W230F	>200
S234R	>200

Structure-based mutants in dimeric EBOV VP30 CTD were assessed for binding to monomeric EBOV NP 600–617 fused to T4 lysozyme using ITC. Mutants for which binding was too weak to be measured with the protein concentrations used (120 μM NP peptide, 1200 μM VP30 CTD) are listed as having equilibrium dissociation constants (K_D_) greater than 200 μM (c-value < 1). See also S2 Fig.

**Fig 6:**
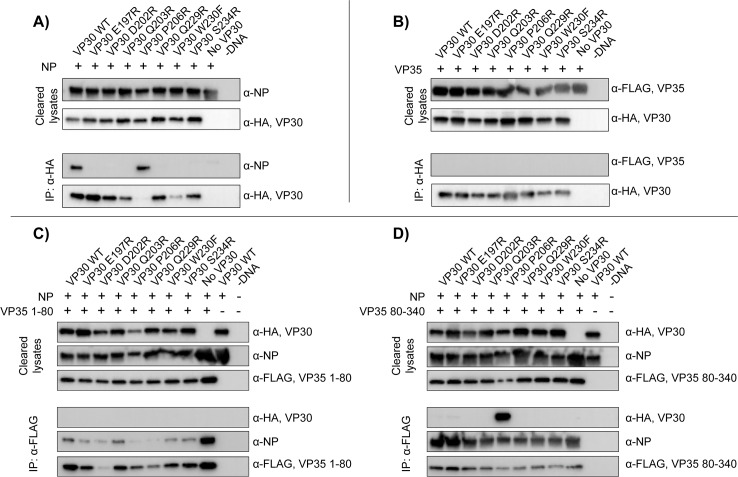
Co-immunoprecipitation of VP30 viral interaction partners. Immunoprecipitation of HA-tagged VP30 and VP30 mutants with A) NP or B) VP35. Immunoprecipitation of VP30 with C) FLAG-tagged VP35 1–80 and monomeric NP or D) with FLAG-tagged VP35 80–340 and oligomeric NP-RNA complexes.

We further examined the interactions of these mutants and NP in the context of full-length proteins using co-immunoprecipitation of NP with HA-tagged VP30 (Fig 6A). Under our stringent wash conditions (50 mM TrisCl pH 8.0, 300 mM NaCl, 2 mM EDTA, 10% glycerol, 1% Igepal CA-630, 2 mM beta-mercaptoethanol) and treatment of lysates with benzonase nuclease to examine protein-protein interactions, only the wild-type VP30 and the VP30 P206R pulled down co-transfected NP. Curiously, VP30 P206R, itself, immunoprecipitated poorly on the anti-HA beads despite its presence in the soluble protein fraction and pulling down a significant amount of NP. This result was highly reproducible and was only observed in the presence of co-transfected NP and not co-transfected VP35 (Fig 6B). These results suggest that the poor pull down of VP30 P206R is a result of its intimate interaction with the highly oligomeric NP and is not due to improper protein folding. As EBOV VP30 has also been reported to interact with VP35 [6] we performed a similar co-immunoprecipitation analysis using FLAG-tagged full-length VP35. For none of the VP30 variants, including the wild-type, do we observe VP35 pulldown with VP30 (Fig 6B). This is congruent with a recent study which showed that the previously observed VP35-VP30 associations are mediated by RNA and are sensitive to nucleases [15].

Non-segmented negative sense RNA virus nucleoproteins are chaperoned as monomers by the viral phosphoproteins (filovirus VP35), prior to their incorporation into the large oligomeric, RNA-bound complexes that are the templates used by the phosphoprotein-polymerase protein complex for RNA synthesis. Having shown that VP30 and VP30 mutants interact with NP, we sought to determine which NP oligomeric form binds VP30. To do this, we separated the NP-binding activities of VP35 into the chaperoning region (VP35 1–80) [16, 21] and the oligomeric C-terminal region (VP35 80–340) thought to be important for VP35’s activity as a polymerase co-factor, as has been shown for other non-segmented negative-sense RNA viruses [16, 28–30]. By pulling down on each of these FLAG-tagged VP35 truncations, we are able to select for either the chaperoned, monomeric NP or the oligomeric, RNA-bound NP. In the case of the chaperoned, monomeric NP, the co-transfection of any of our VP30 constructs, including wild-type, greatly diminishes the pulldown of NP with VP35 1–80. None of these VP30 constructs, nor wild-type VP30, pulled down with the VP35 1–80 chaperoned NP (Fig 6C). For the oligomeric, RNA-bound NP selected by VP35 80–340, the only VP30 variant to pull down is the P206R mutant, consistent with the higher affinity of this mutant for NP (Fig 6D). That wild-type VP30 did not pull down with NP in the presence of VP35 80–340, as it did with NP alone (Fig 6A), is likely reflective of the stringent washes used for the pulldown, the weaker affinity of the wild-type VP30 compared to VP30 P206R, and the less robust isolation of secondary interaction partners by co-immunoprecipitation analysis.

**Fig 7:**
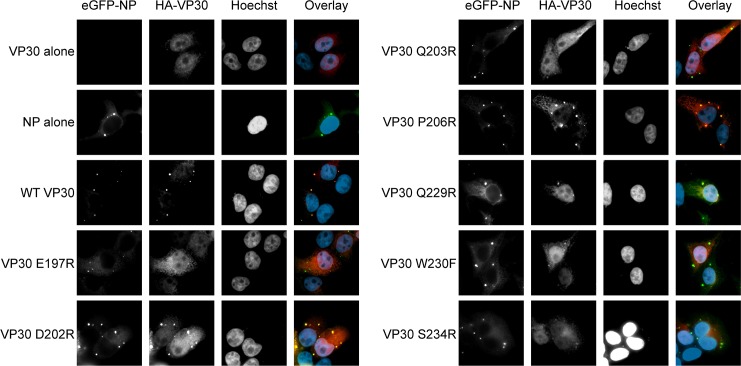
Localization of VP30 to NP inclusions. eGFP-EBOV NP was cotransfected with HA-tagged EBOV VP30 or VP30 mutants DNA constructs into 293T cells. VP30 was detected with a mouse anti-HA primary antibody and a goat anti-mouse, Alexa Fluor 647-conjugated secondary antibody. Nuclei were stained with Hoechst 33342. EBOV NP forms punctate inclusions in transfected cells.

To determine the effects of the EBOV VP30 mutants on binding to NP in cells, we used fluorescence analysis of transfected cells (Fig 7). On its own, the oligomeric NP forms large intracellular inclusions. In virus-infected cells, these inclusions are the sites of viral RNA synthesis [31, 32]. Though ordinarily presenting diffuse cytoplasmic localization, VP30 and VP35 localize to these inclusions when co-expressed with NP [32, 33]. Our fluorescence analysis of the VP30 mutants parallels the ITC data where wild-type VP30, D202R and P206R robustly localize to the NP inclusions, Q229R shows a less robust localization, and E197R, Q203R, W230F and S234R are considerably more diffuse. These data also support the co-immunoprecipitation data above by confirming that VP30 interacts with the oligomeric, RNA-bound NP in the punctate inclusions and not a monomeric form of NP [34].

**Fig 8:**
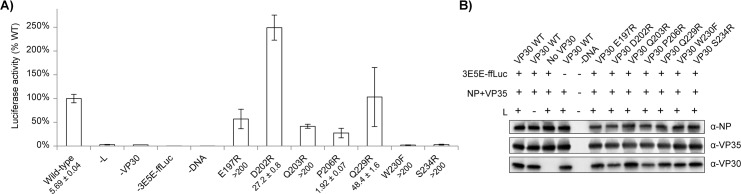
Mutation of the VP30 NP-binding site alters replicase activity. A) Mini-replicon activities are presented as a percentage of the firefly luciferase activity observed with wild-type VP30. Dissociation constants observed in ITC experiments are listed for each mutant. B) Western blot for NP, VP35 and VP30 of cleared lysates from cells transfected with the mini-replicon system, confirming similar levels of expression compared for VP30 mutants to wild-type.

To complement this binding and interaction data, we also assessed the activity of the VP30 mutants in the EBOV mini-replicon assay, which tests their ability to support RNA synthesis (Fig 8). Surprisingly, the firefly luciferase activities from the mini-replicon assays display a complex pattern when compared to VP30-NP interaction affinities. The VP30 P206R mutant, which possesses higher affinity for NP, shows less mini-replicon activity. Conversely, the two VP30 mutants with decreased affinities (D202R and Q229R) show wild-type or better mini-replicon activity. The four VP30 mutants with affinities too low to be assessed in the ITC experiment showed reduced or background levels of mini-replicon activity.

**Fig 9:**
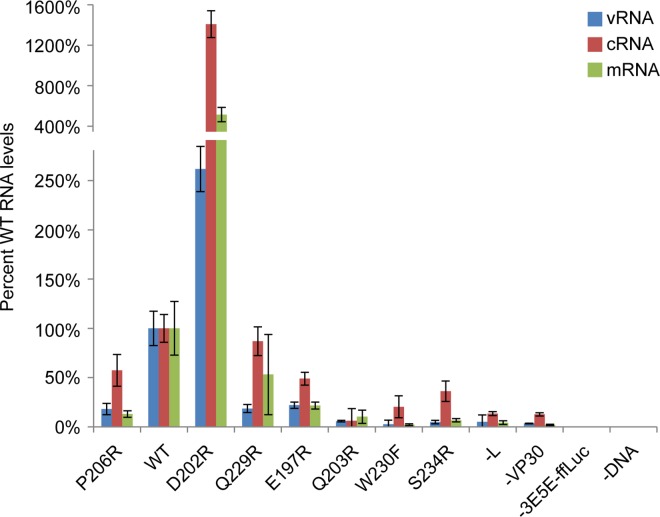
Analysis of RNA levels shows the VP30-NP interaction to be a general modulator of RNA synthesis activity. RNA from mini-replicon transfected cells was reverse transcribed using primers specific for vRNA (blue), cRNA (red) or mRNA (green). Quantitative PCR was performed with primers amplifying the firefly luciferase ORF common to all RNA types. VP30 mutants are indicated and are arranged from high affinity (left) to low affinity (right) binders. Wild-type (WT) and negative controls (-L, -3E5E-ffLuc, -DNA) are presented for comparison. Data are normalized to the wild-type control samples.

In infected cells, viral replication generates three distinct populations of RNA: genomic viral RNA (vRNA), complementary RNA (cRNA) and messenger RNA (mRNA). The negative-sense vRNA acts as a template for the synthesis of positive-sense cRNA and mRNA. The cRNA, in turn, acts as a template for the synthesis of additional vRNA, producing additional copies of the viral RNA genome. One of the proposed functions of VP30 is regulation of the balance of various viral RNA species during viral replication. Mutagenesis of VP30 can alter the ratios of vRNA, cRNA and mRNA in infected and transfected cells [6, 7]. Thus, we sought to better define the functional consequences of altering VP30-NP interactions with respect to each of these RNA species, in the hope of better understanding the specific processes the VP30-NP interaction mutants would impact. We evaluated the RNAs produced from mini-replicon transfected cells using qRT-PCR (Fig 9). Although we observe differences in the relative levels of vRNA, cRNA, and mRNA for the different VP30 mutants, the most striking effect of the VP30 mutants is on the overall amounts of all RNAs produced, which correlates with the firefly luciferase activities. These data suggest that the VP30-NP interaction plays a primary role in the regulation of overall RNA synthesis activity by the viral nucleocapsid complexes.

Particularly striking are the high levels of viral RNAs seen with the VP30 D202R mutant, especially for the cRNA. However, the amount of mRNA for the other VP30s appears to follow the relative amount of vRNA suggesting that the transcriptional activity is relatively unaltered (at most ~2-fold) by changes in the VP30-NP interaction. While we found that VP30 is required for transcription, we were surprised that in the absence of VP30, neither vRNA nor cRNA is produced, contrary to previous reports that VP30 is not necessary for viral replication [3, 35]. These previous studies used a mini-replicon system using a different vector backbone for expression of the ebolavirus proteins, different plasmid ratios and different reporter genes. However, these systems are conceptually identical and these minor differences are not expected to account for differing results. Our finding that VP30 is essential for viral RNA transcription and replication is supported by our data from the VP30 W230F and S234R mutants which showed undetectable binding in the ITC analysis, minimal luciferase activity in the mini-replicon system, and RNA profiles similar to those of the–L and–VP30 negative controls.

## Discussion

In this work, we have identified the region of filovirus NPs necessary and sufficient for interaction with VP30. We broadened our initial screening for the EBOV NP-VP30 interaction to confirm that this interaction also occurs in SUDV and MARV, highlighting the conserved nature of the NP-VP30 interaction and suggesting an important role for this interaction in the filovirus life cycles. Our crystal structures of the EBOV and MARV NP-VP30 complexes have revealed the molecular determinants of these interactions and have allowed us to structurally design mutants specifically targeting the VP30 and NP interfaces.

**Fig 10:**
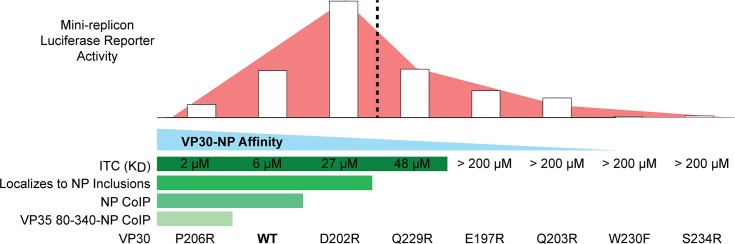
The gradient of VP30-NP interaction affinities shows two phases of RNA synthesis activity. Increasing affinity (decreasing K_D_) of the VP30-NP interaction (blue) from wild-type (WT), as assessed by multiple assays (see Table 2, Figs 4 and 7), represses viral RNA synthesis activity (pink) (Figs 8 and 9) while mildly decreasing affinity activates viral RNA synthesis. A transition occurs beyond the affinity of the VP30 D202R mutant (dashed line) such that further reductions in affinity result in diminished RNA synthesis activity reflecting the essential nature of the VP30-NP interaction to the RNA synthesis complex.

We used our structure-based mutagenesis of the EBOV NP-binding site on VP30 to assess the binding of NP to VP30 using a myriad of assays (Fig 10). The ITC data indicate a range of mutant VP30 CTD affinities for the NP 600–617 peptide, including some mutants for which NP binding was not apparent. These data are supported by the fluorescence data, which show VP30 localization to NP inclusions for only those VP30 constructs with high affinity binding to NP in ITC. Those VP30 CTD mutants that were not observed to bind NP 600–617 in ITC (E197R, Q203R, W230F and S234R) could possibly bind NP if higher protein concentrations were used in the ITC assay. However, further increasing the protein concentrations of the VP30 CTD and NP 600–617 beyond those used in the ITC experiments presented is not feasible. A further limitation to the interpretations of the ITC data is that it uses highly truncated, dimeric VP30 and monomeric NP to accurately measure the affinity of the molecular interaction. Although VP30-NP binding was not observed for some VP30 mutants in ITC experiments, the oligomerization of full-length VP30 and NP is expected to increase the avidity of binding as well as to increase the NP-affinity differences among the VP30 mutants. Analogously, in co-immunoprecipitation experiments, only wild-type VP30 and VP30 P206R could be shown to interact with NP despite measurable interactions between the NP 600–617 peptide and VP30 CTD mutants D202R and Q229R in the ITC assay. This indicates that while assays such as ITC, immunofluorescence and co-immunoprecipitation can reveal protein-protein interactions, they sample a select range of interaction affinities and may exclude biologically relevant interactions.

It was previously concluded that the VP30-NP interaction was not necessary for viral transcription based on data showing that a VP30 E197A mutant maintained activity in a mini-replicon assay, but did not pull down NP in co-immunoprecipitation experiments, although the VP30 E197A mutant did partially co-localize with NP inclusions in cells [6, 11]. The data we present here demonstrate that modifying the affinity of VP30 for NP has differing effects on the outcome of mini-replicon reporter assays, and that these affinities are differentially assessed by assays such as co-immunoprecipitation, ITC, and co-localization in immunofluorescence analysis. A possible explanation for the observed mini-replicon activity for VP30 mutants that are negative for binding to NP, as assessed by assays such as ITC, immunofluorescence or co-immunoprecipitation, is that even a weak or transient interaction of VP30 with NP is capable of promoting RNA synthesis activity. Examples of weak VP30-NP binding permitting RNA-synthesis activity are the previously characterized VP30 E197A mutant [11] and the VP30 Q229R mutant, which though showing a weak affinity in ITC experiments, possesses a wild-type level of RNA synthesis. This is in contrast to the VP30 W230F and S234R mutants that showed neither NP binding in *in vitro* assays nor activity in mini-replicon assays and may represent complete knockouts for the VP30-NP interaction.

Comparison of the mini-replicon assay luciferase activities and qRT-PCR with the mutant VP30-NP interactions as assessed by ITC, co-immunoprecipitation and fluorescence localization of VP30 to NP inclusions reveals an interesting and unexpected trend (Fig 10). Along the gradient of high-affinity to low-affinity VP30-NP interactions, there appears to be a transition in the effects of VP30-NP affinity on RNA synthesis, belying at least two functions for the VP30-NP interaction in viral RNA synthesis. Beginning on the high-affinity end of the gradient, high-affinity NP-binding VP30 P206R mutant actually decreases viral RNA synthesis activity and modestly weakening the VP30-NP interaction increases RNA synthesis activity. The RNA synthesis activity reaches a maximum for the VP30 D202R mutant and then transitions such that further decreases in the VP30-NP affinity decrease RNA synthesis. The first phase of this affinity gradient suggests that the VP30-NP interaction is inhibitory for RNA synthesis while the second phase is reflective of the essential nature of VP30 for RNA synthesis as we see in our -VP30 control samples.

The VP30-NP binding and RNA synthesis activity data suggests multiple functions for the VP30-NP interaction in the filovirus life cycle. A possible mechanism accounting for the two phases of RNA synthesis activity with VP30-NP affinity is that VP30 serves as a positive RNA synthesis co-factor and also stabilizes the nucleocapsid. High-affinity VP30-NP interactions may over-stabilize the nucleocapsid and restrict access of the viral polymerase to the viral genome. Conversely, modestly decreasing the affinity of the VP30-NP interaction would allow greater access of the polymerase to the viral genome. However, further decreases in VP30-NP affinity strongly impact VP30’s function as an RNA synthesis activator, impairing polymerase function. The strength of this hypothesis remains to be tested by future studies of nucleocapsid assembly, stability and polymerase function.

Our data show that EBOV, SUDV and MARV VP30s recognize a short peptide in the C-terminal region of their respective NPs. In addition, we have described the molecular interaction of these components with crystal structures and shown this interaction to be essential for viral RNA synthesis. Although the VP30-NP interaction is essential for RNA synthesis, it appears that this interaction serves to reduce the RNA synthesis activity and that to an extent, modestly destabilizing this interaction gives a boost to viral RNA synthesis (Figs 8 and 9). This suggests that the VP30-NP interaction tunes the overall RNA synthesis activity to a level ideal for viral infection, rather than maximal RNA synthesis.

Because the VP30-NP interaction is essential and highly conserved and because the strength of this interaction modulates RNA synthesis activity, therapeutic drugs either blocking or stabilizing this interaction may be efficacious in treating filovirus infections. Escape mutations arising in VP30 or NP in response to such therapeutic drugs are predicted to have disregulated RNA synthesis activity possibly resulting in attenuated viruses.

## Materials and Methods

### Antibodies

Full length EBOV NP was detected by Western blotting using human antibody KZ51 (gift from Dennis Burton). EBOV NP truncations and mutants were detected by Western blotting using rabbit anti-2A peptide antibody ABS31 (Millipore). EBOV VP30 and VP30 mutants for the mini-replicon experiments were detected by Western blotting with a polyclonal rabbit anti-VP30 antibody (gift from Yoshihiro Kawaoka and Pete Halfmann). EBOV VP35 in the mini-replicon experiments was detected by Western blotting using a mouse anti-VP35 antibody (gift from Victor Volchkov). In co-immunoprecipitation experiments, HA-tagged VP30 proteins were detected by Western blotting using a rabbit anti-HA antibody (Rockland Immunochemicals) and FLAG-tagged VP35 proteins were detected using a rabbit anti-FLAG antibody (Cell Signaling Technology). Secondary antibodies used for Western blotting were goat anti-mouse or -rabbit or -human Fc HRP conjugates (Thermo Fisher). For immunofluorescence, HA-tagged VP30 was detected with 16B12 (BioLegend) and a goat anti-mouse Alexa 647 conjugate (Thermo Fisher).

### Protein Expression and Purification

NP and VP30 protein constructs for bacterial expression were cloned into pET46 (Novagen), which contains an N-terminal hexahistidine tag and enterokinase cleavage site. For producing biotinylated NP peptides, pET46 was modified to additionally include an N-terminal AviTag. Bacterially expressed proteins were produced in Rosetta2 pLysS E. coli (Novagen). 1 L cultures were grown at 37°C to an OD_600_ of 0.4 and induced with isopropyl-β-D-1-thiogalactoside (IPTG, 0.5 mM final concentration). The temperature was reduced to 25°C and expression was carried out overnight. Bacteria were harvested by centrifugation and resuspended in Ni-NTA binding/wash buffer (50 mM HEPES pH 7.4, 300 mM NaCl, 30 mM imidazole, 2mM BME). Resuspended cells were lysed using a Microfluidizer M110-P (Microfluidics). Lysates were cleared by centrifugation at 25,000×g for 30 minutes and then filtered through a 0.22 μm filter. 2 mL of Ni-NTA Agarose (Qiagen) was added to the lysates and allowed to incubate for 30 minutes. Agarose beads were collected and washed twice with 20 mL of Ni-NTA binding buffer in a gravity column format. Bound proteins were eluted with the same buffer containing 300 mM imidazole. Proteins were concentrated by ultrafiltration (Amicon, Millipore) prior to size exclusion chromatography (Superdex 200, GE Life Sciences) in 50 mM HEPES pH 7.4, 300 mM NaCl, 5mM BME. For the expression of biotinylated NP peptides, NP-expressing plasmids were co-transformed with a plasmid carrying E. coli biotin ligase and 1 L cultures were supplemented with 13 mg of D-biotin at the time of expression induction. These peptides were purified identically to other expressed proteins with Superdex 75 used for size exclusion chromatography.

### Biolayer Interferometry

For biolayer interferometry (BLI) screening of EBOV NP-VP30 protein interactions, all proteins were prepared in 1X BLI buffer (50 mM HEPES pH 7.4, 300 mM NaCl, 5 mM BME, 10μg/mL bovine serum albumin and 0.002% Tween-20). All BLI experiments were performed using an Octet Red (Forte Bio) and streptavidin coated biosensors. Biosensors were equilibrated in 1X BLI buffer for 5 minutes prior to the start of the experiment. Biosensors were successively dipped into wells containing 1X BLI buffer (60 s), 50 μg/mL biotinylated EBOV NP peptides (120 s), 1X BLI buffer (120 s), 10 μM VP30 CTD (180 s) and 1X BLI buffer (180 s). Because EBOV VP30 CTD is dimeric, complicating quantitative analysis, these experimental data were used qualitatively.

### Isothermal Titration Calorimetry

Expressed and purified proteins were dialyzed overnight into 25 mM HEPES pH 7.4, 300 mM NaCl, 5 mM BME. EBOV NP 360–739 constructs and NP 600–617 fused to T4-lysozyme were concentrated to 120–140 μM as determined by UV absorbance at 280 nm. VP30 CTD constructs were concentrated to 1,200–1,600 μM. NP constructs were loaded into the calorimeter cell and VP30 constructs were loaded into the titration syringe. All experiments were carried out using an Auto-iTC200 (Malvern) operating at 25°C and 1000 rpm unless otherwise noted. ITC data was processed with Origin software. Reported dissociation constants and errors are the average and standard deviation of three replicates.

### Viral Mini-genome Assays

EBOV mini-genome assays were performed similar those previously described [20]. Briefly, HEK 293T cells (gift from Dr. Dennis Burton) were grown to 80–90% confluency in DMEM, 5% FBS, 50 U/mL penicillin and 50 μg/mL streptomycin in 12-well format. Cells were transfected with 2 μg of EBOV L-pCAGGS, 0.75 μg VP35-2A-NP-pCAGGS, 0.25 μg VP30-pCAGGS, 0.5 μg T7 Polymerase-pCAGGS, 0.5 μg 3E5E-ffLuc and 0.1 μg Renilla luciferase-pCMV (Promega) mixed with TransIT-LT1 (Mirus Bio) in a 3:1 ratio (μL/total DNA μg). Transfected cells were harvested after 48 hours. For luciferase activities, cells were washed with phosphate-buffered saline and lysed with 1X Passive Lysis Buffer (Promega). Lysates were flash-frozen and thawed, and then cleared by centrifugation prior to measuring luciferase activities using the Dual-Luciferase Assay Reporter System (Promega) read on a Spark 10M (Tecan) using injectors. Luciferase activity for each transfection is reported relative to wild-type control and normalized for differences in transfection efficiency using the Renilla luciferase activity as an internal control. Transfections were performed at least three times.

### Co-immunoprecipitation

For VP30 co-immunoprecipitation of NP or VP35, 0.5 μg of N-terminally HA-tagged VP30-pDisplay or mutants were cotransfected in HEK 293T cells with 0.5 μg of either full-length NP or full-length N-terminally FLAG-tagged VP35 mixed with 1mg/mL PEI MAX (Polysciences) in a 3:1 (μL/total DNA μg) in 12-well plates. For VP30 co-immunoprecipitation of NP and truncated VP35, 0.5 μg of HA-tagged VP30-pDisplay was combined with 0.25 μg of NP-pDisplay and 0.25 μg of either C-terminally tagged VP35 1-80-pDisplay or N-terminally tagged VP35 80-340-pDisplay. Transfected cells were harvested after 48 hours. Cells were washed with 100 μL TBS and lysed in Cell Lysis Buffer (50 mM TrisCl pH 8.0, 300 mM NaCl, 2 mM EDTA, 10% glycerol, 1% Igepal CA-630, 2 mM BME, 250 U/mL benzonase nuclease (Novagen)) for 10 minutes. Lysates were cleared by centrifugation. Lysate supernatants were mixed with 25 μL anti-HA agarose beads (3F10, Roche) or 25 μL anti-FLAG agarose beads (M2, Sigma) and incubated at 4°C for 30 minutes with agitation. Beads were then washed three times with 800 μL of Cell Lysis Buffer (without benzonase) and then mixed with 30 μL of SDS-PAGE sample loading buffer (6% SDS, 30% glycerol, 180 mM TrisCl pH 6.8, 0.0125% bromophenol blue) and boiled for 1 minute before loading gels for Western blotting analysis.

### Immunofluorescence Analysis

HEK 293T cells were grown on poly L-lysine coated coverslips. Cells were transfected with 0.25 μg of N-terminally HA-tagged VP30-pDisplay and 0.25 μg of eGFP-NP-pDisplay with TransIT-LT1 (MirusBio) in a 3:1 ratio (μL/μg DNA). 24 hours post-transfection, coverslips were washed in phosphate buffered saline (PBS) and cells were fixed with 2% paraformaldehyde for 20 minutes. Coverslips were washed three times with PBS and then quenched and permeabilized (0.5% Triton X-100, 20 mM glycine in phosphate buffered saline) for 20 minutes. Coverslips were washed three times with PBS, blocked (2% bovine serum albumin (BSA) in PBS) for 30 minutes and then incubated with a 1/500 dilution of anti-HA antibody in 1% BSA and PBS for 45 minutes. Coverslips were washed three times in PBS and then incubated with a 1/2000 dilution of secondary antibody, goat anti-mouse IgG conjugated to Alexa Fluor 647 in 1% BSA and PBS for 45 minutes. Coverslips were washed with three times with PBS and then stained with 1 μg/mL Hoechst 33342 in PBS for 5 minutes and then washed twice with PBS and once with water. Coverslips were mounted on glass slides using Prolong Gold (Invitrogen). Slides were imaged on a Nikon TE2000 epifluorescence microscope and images were collected and processed with Metamorph software (Molecular Devices).

### Quantitative Reverse-Transcriptase Polymerase Chain Reaction

In addition to measuring luciferase activities of mini-genome transfected cells, select reactions were repeated and assessed using qRT-PCR to examine relative amounts of vRNA, cRNA and mRNA. Cells were transfected as above. After 48 hours, RNA was harvested from the cells using the RNeasy Plus Mini Kit (Qiagen). cDNA was generated using 500 ng of RNA and the SuperScript III First-Strand Synthesis System (Invitrogen) using primers specific for vRNA (GACAAATTGCTCGGAATCACAAAATTCC), cRNA (CACGCAGGGAGAGAGGCTAAATATAG) or mRNA (poly(dT_20_)). qPCR was performed using primers directed to amplify firefly luciferase (GCTATTCTGATTACACCCGAGG, TCCTCTGACACATAATTCGCC), GAPD (AAGGTGAAGGTCGGAGTCAA, AATGAAGGGGTCATTGATGG) or RPLP0 (GCGACCTGGAAGTCCAACTA, ATCTGCTTGGAGCCCACAT). The reactions were run using 2X SYBR Select Master Mix (Applied Biosystems) and a CFX384 Touch Real-Time PCR Detection System (Bio-Rad). Data was analyzed using Bio-Rad CFX Manager using the GAPD and RPLP0 as reference controls. A serial dilution of 3E5E-ffLuc plasmid was included to assess the efficiency of amplification of the firefly luciferase cDNAs. qPCR reactions were repeated four times.

### Crystallization

Due to the low affinity of the EBOV NP 600–617 for VP30 CTD, these proteins were cloned as a fusion protein for structural studies using X-ray crystallography. EBOV NP 600–627 was fused to the N-terminus of VP30 CTD and purified as described above. Crystals were grown using 0.3 μL of 20 mg/mL protein mixed with 0.3 μL of 2.2 M ammonium sulfate, 100 mM sodium acetate pH 4.9 in a sitting drop, vapor diffusion setup. Crystals were cryo-protected in 3.15 M ammonium sulfate, 50 mM sodium acetate pH 5.0 and 5% glycerol prior to cryo-cooling the crystals in liquid nitrogen. MARV NP 552-579-VP30 146–281 fusion protein crystallized in 20% isopropanol, 18% PEG 4000, 0.1 M sodium citrate pH 5.6 using 0.2 μL 24.7 mg/mL protein mixed with 0.2 μL mother liquor. MARV crystals were cryoprotected with 20% 2-methyl 2,4-pentanediol in mother liquor prior to cryo-cooling in liquid nitrogen.

### Data Collection, Processing and Refinement

Diffraction data were collected at the National Institute of General Medical Sciences and National Cancer Institute Structural Biology Facility (GM/CA) at the Advanced Photon Source, Argonne National Lab at beamline 23ID-D. Diffraction data was processed with XDS [36] and merged with AIMLESS [37]. The EBOV NP-VP30 structure was determined using molecular replacement as implemented in Phaser [38] and 2I8B.pdb [11] as a search model, while for the MARV NP-VP30 molecular replacement, the 5DVW.pdb [26] was used as a search model. The models were rebuilt in Coot [39] and refined using Phenix [40] and Refmac [41]. Analysis of the protein interaction surface was done in PISA [42].

### Accession Numbers

Coordinates and structure factors for the EBOV NP-VP30 complex, 5T3T.pdb, and the MARV NP-VP30 complex, 5T3W.pdb, are deposited in the Protein Data Bank (www.rcsb.org). GenBank accession numbers for proteins used in this study: EBOV NP: AAD14590.1, EBOV VP30: AAD14587.1, SUDV NP: AGB56675.1, SUDV VP30: YP_138525.1, MARV NP: YP_001531153.1, MARV VP30: YP_001531157.1, EBOV VP35: NP_066244.1, EBOV L: NP_066251.1 (http://www.ncbi.nlm.nih.gov/).

## Supporting Information

S1 Fig The EBOV VP30 CTD binds a peptide in the C-terminal region of NP. Biotinylated NP peptides are loaded onto streptavidin-coated biosensors and then presented to VP30 CTD during biolayer interferometry. A) BLI experiments identify a large NP peptide region, 560–617, in the NP C-terminal region as interacting with NP. B) Within NP 560–617, VP30 recognizes shorter NP peptides suggesting that the VP30 binding site lies in NP 600–617.
(TIF)

S2 Fig VP30 CTD recognizes the NP 600–617 peptide. This ITC data shows the interaction of the VP30 CTD with the NP C-terminal region and NP peptide (600–617). These data also show that VP30 CTD does not interact with T4 lysozyme or an NP C-terminal region in which the conserved binding site has been mutated (upper panel). Mutations to the VP30 binding site result in altered affinities for the NP peptide (lower panels).
(TIF)

S3 Fig ITC yields thermodynamic parameters for the VP30-NP binding event. The slope of the enthalpy change upon binding with temperature yields the heat capacity change (ΔC_p_) [23]. The x-intercept of the entropy change upon binding with temperature yields the extrapolated temperature at which the entropy change is zero (T_S_) [24].
(TIF)
