# Supplementary figures and images for: The Ebola Virus VP30-NP Interaction Is a Regulator of Viral RNA Synthesis
Source: PLoS Pathog. 2016 Oct 18;12(10):e1005937. doi: 10.1371/journal.ppat.1005937 (PMC5068707; doi:10.1371/journal.ppat.1005937)

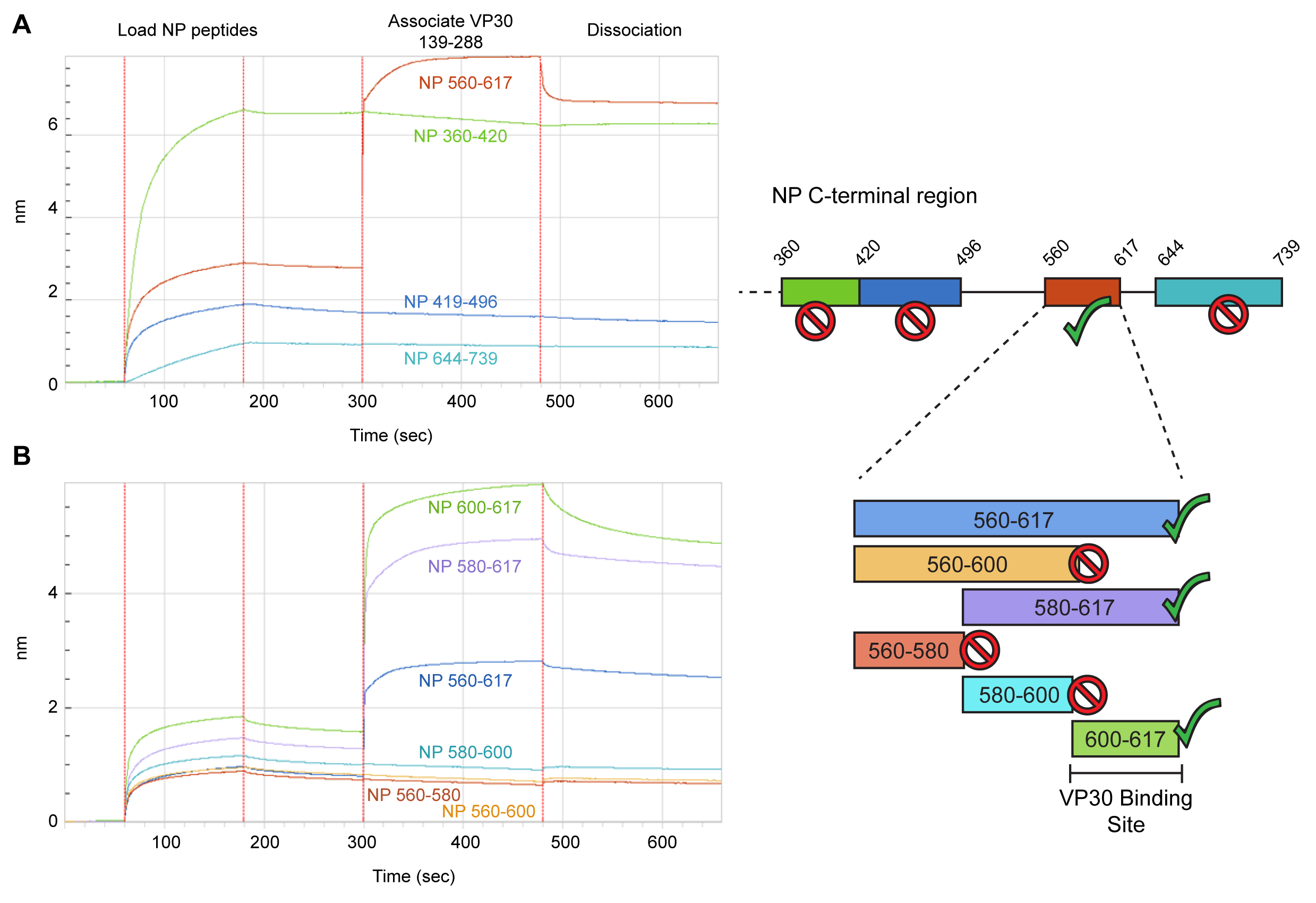

Supplement: S1 Fig — Biotinylated NP peptides are loaded onto streptavidin-coated biosensors and then presented to VP30 CTD during biolayer interferometry. A) BLI experiments identify a large NP peptide region, 560–617, in the NP C-terminal region as interacting with NP. B) Within NP 560–617, VP30 recognizes shorter NP peptides suggesting that the VP30 binding site lies in NP 600–617. (TIF) [file ppat.1005937.s001.tif]

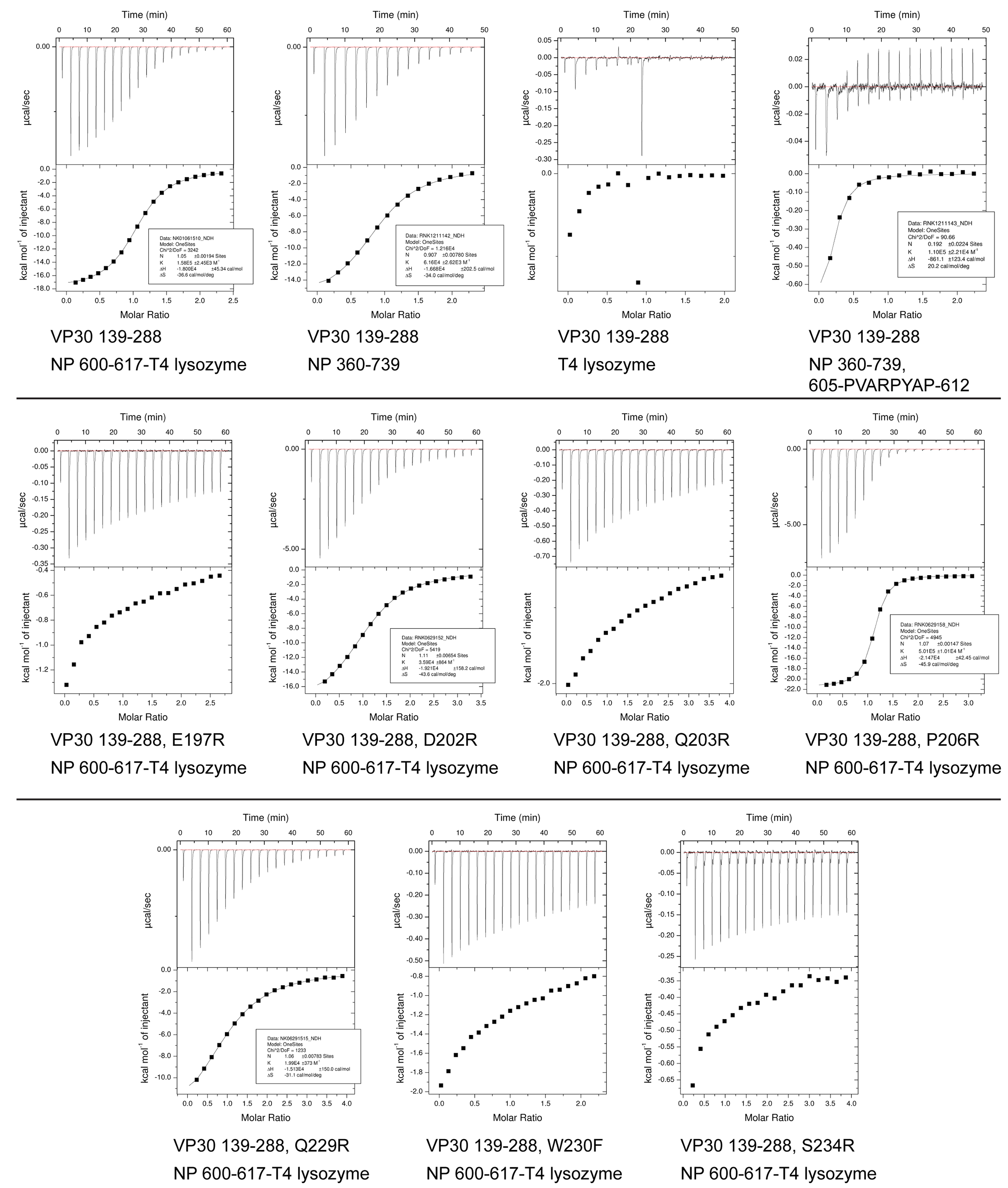

Supplement: S2 Fig — This ITC data shows the interaction of the VP30 CTD with the NP C-terminal region and NP peptide (600–617). These data also show that VP30 CTD does not interact with T4 lysozyme or an NP C-terminal region in which the conserved binding site has been mutated (upper panel). Mutations to the VP30 binding site result in altered affinities for the NP peptide (lower panels). (TIF) [file ppat.1005937.s002.tif]

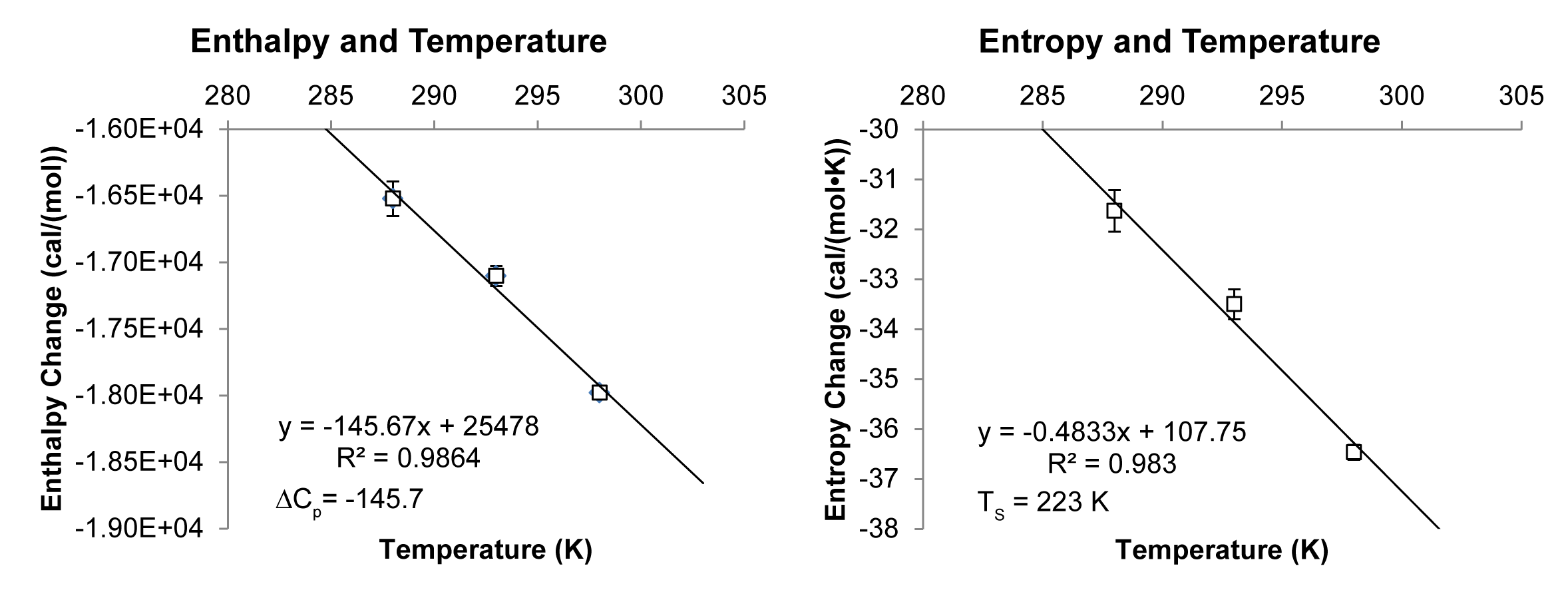

Supplement: S3 Fig — The slope of the enthalpy change upon binding with temperature yields the heat capacity change (ΔCp) [23]. The x-intercept of the entropy change upon binding with temperature yields the extrapolated temperature at which the entropy change is zero (TS) [24]. (TIF) [file ppat.1005937.s003.tif]
